# Supplementary figures and images for: Key epigenetic and signaling factors in the formation and maintenance of the blood-brain barrier
Source: eLife. 2024 Dec 13;12:RP86978. doi: 10.7554/eLife.86978 (PMC11643625; doi:10.7554/eLife.86978)

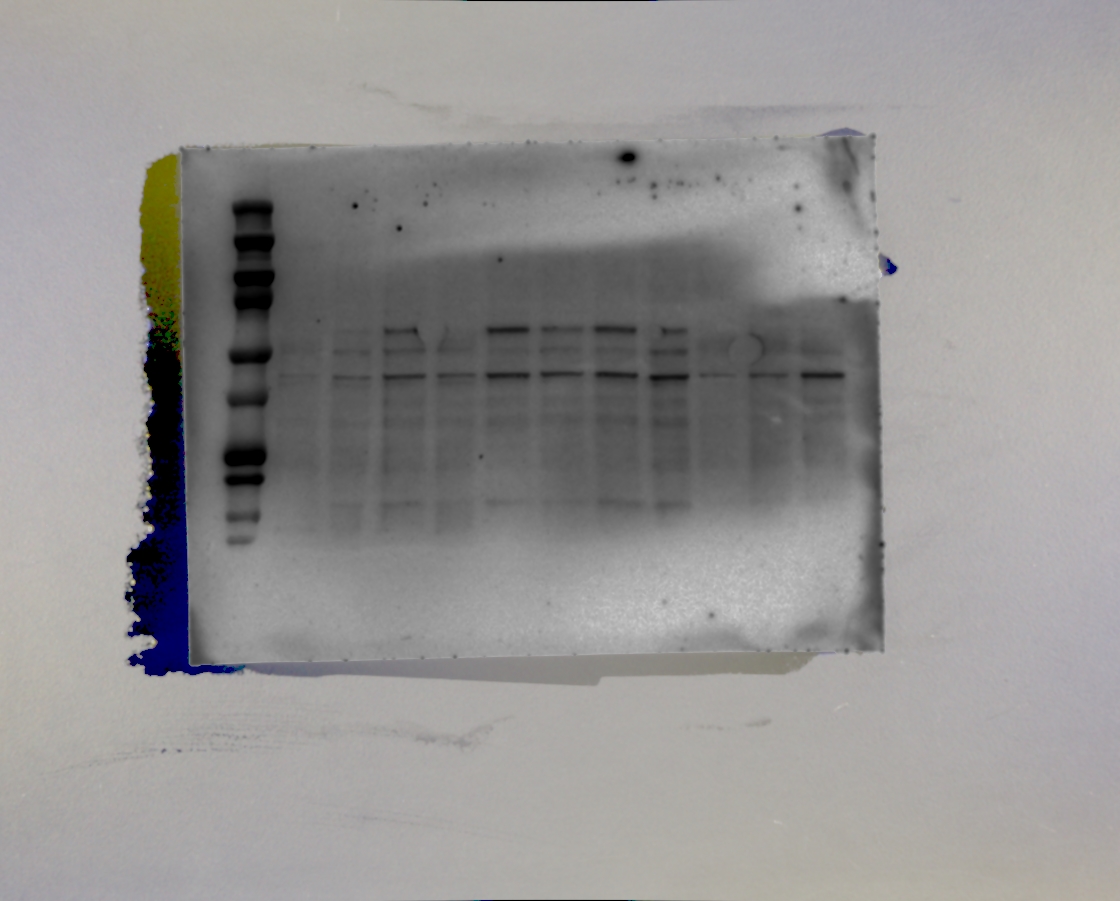

Supplement: Figure 2—figure supplement 1—source data 1. [file elife-86978-fig2-figsupp1-data1.zip › beta actin.jpg]

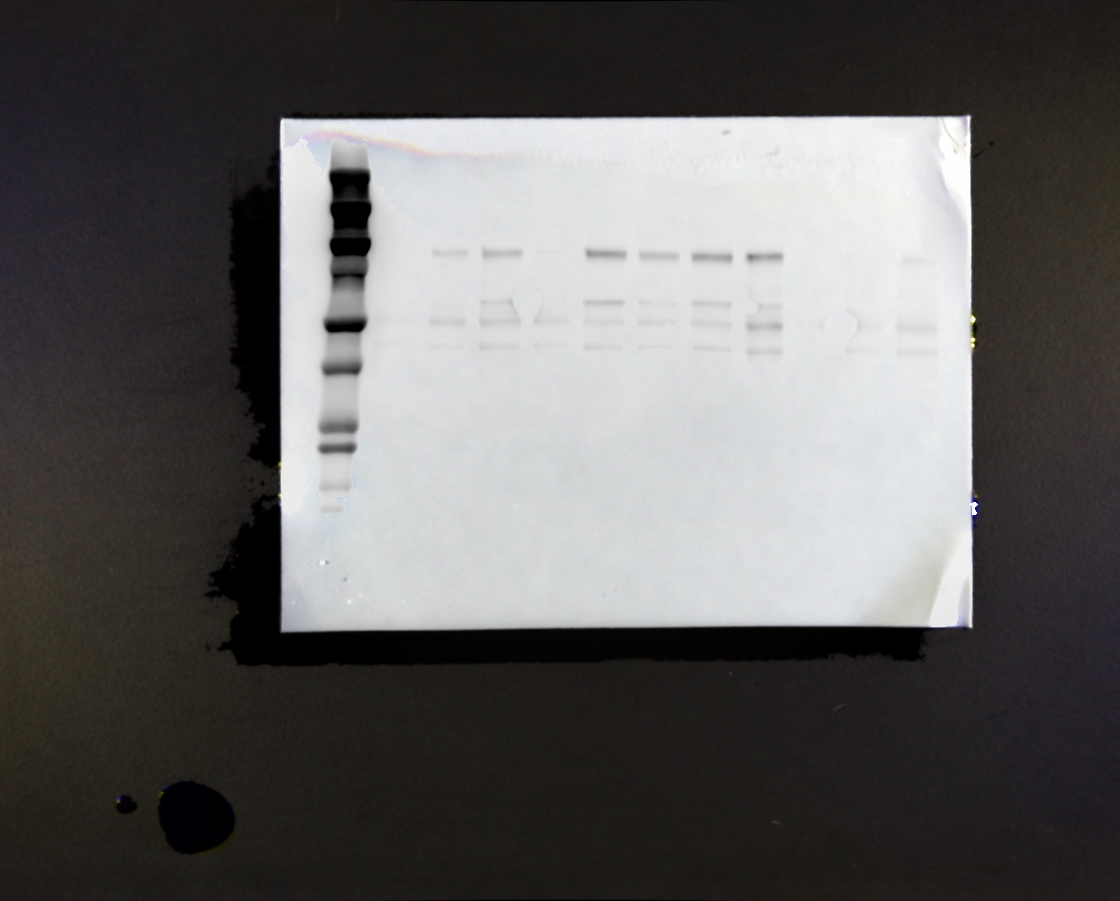

Supplement: Figure 2—figure supplement 1—source data 1. [file elife-86978-fig2-figsupp1-data1.zip › EZH2.jpg]

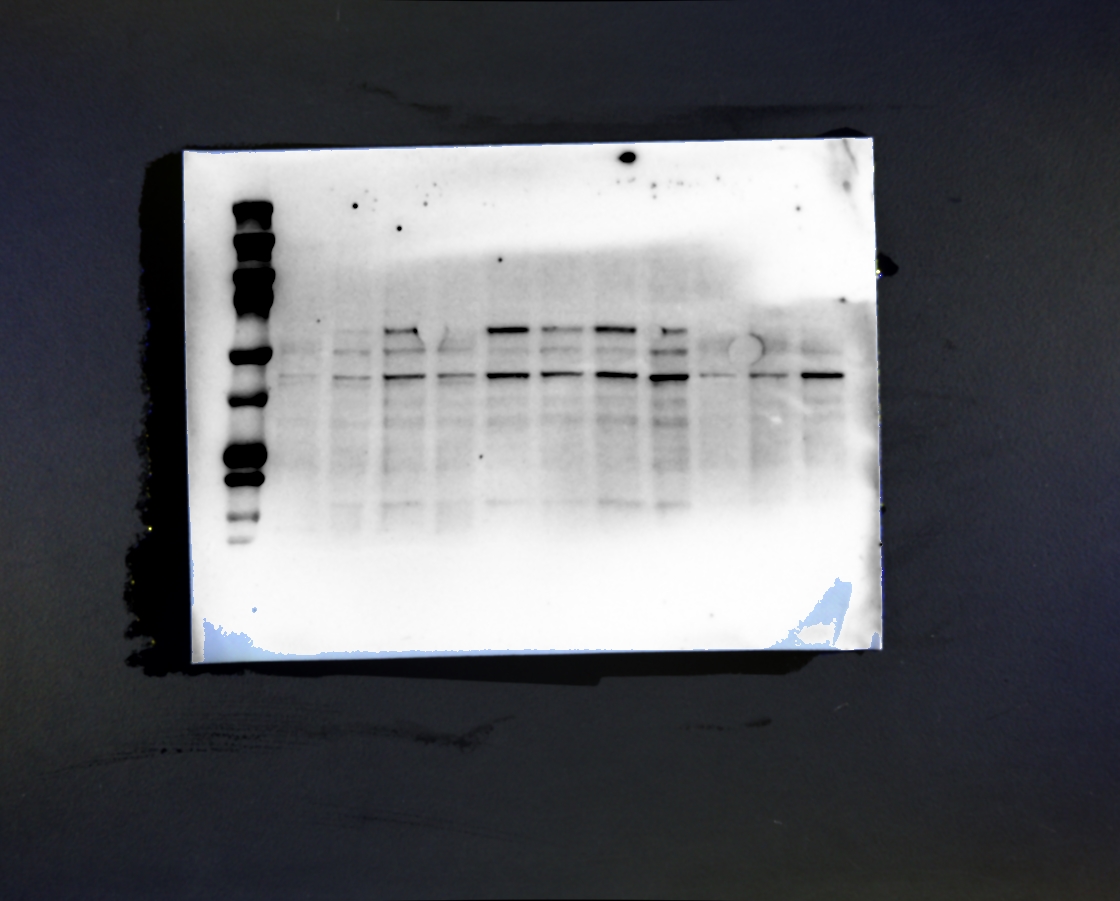

Supplement: Figure 2—figure supplement 1—source data 1. [file elife-86978-fig2-figsupp1-data1.zip › HDAC2.jpg]
