# Supplementary material for: Key epigenetic and signaling factors in the formation and maintenance of the blood-brain barrier
Source: eLife. 2024 Dec 13;12:RP86978. doi: 10.7554/eLife.86978 (PMC11643625; doi:10.7554/eLife.86978)
Supplement: Figure 2—figure supplement 1—source data 2. [file elife-86978-fig2-figsupp1-data2.zip › Fig supplement 2 B,E.pdf]

Figure 2 figure supplement 2B, E, Source Data . Original membranes corresponding to Figure Figure 2 figure supplement 2B, Rainbow molecular weight markers were employed. All treatment conditions are blotted in single gel. Blots probed for b-actin,HDAC2 and EZH2 are shown respectively.

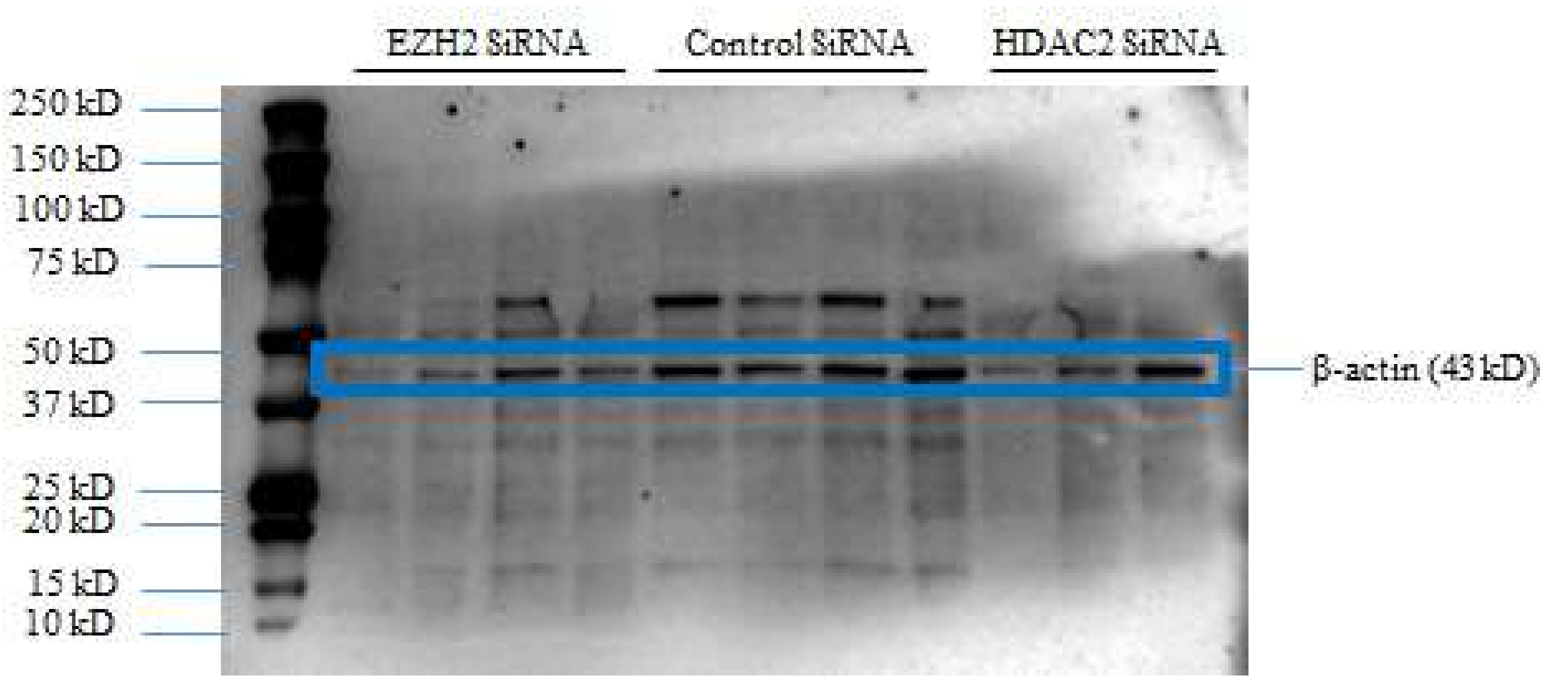

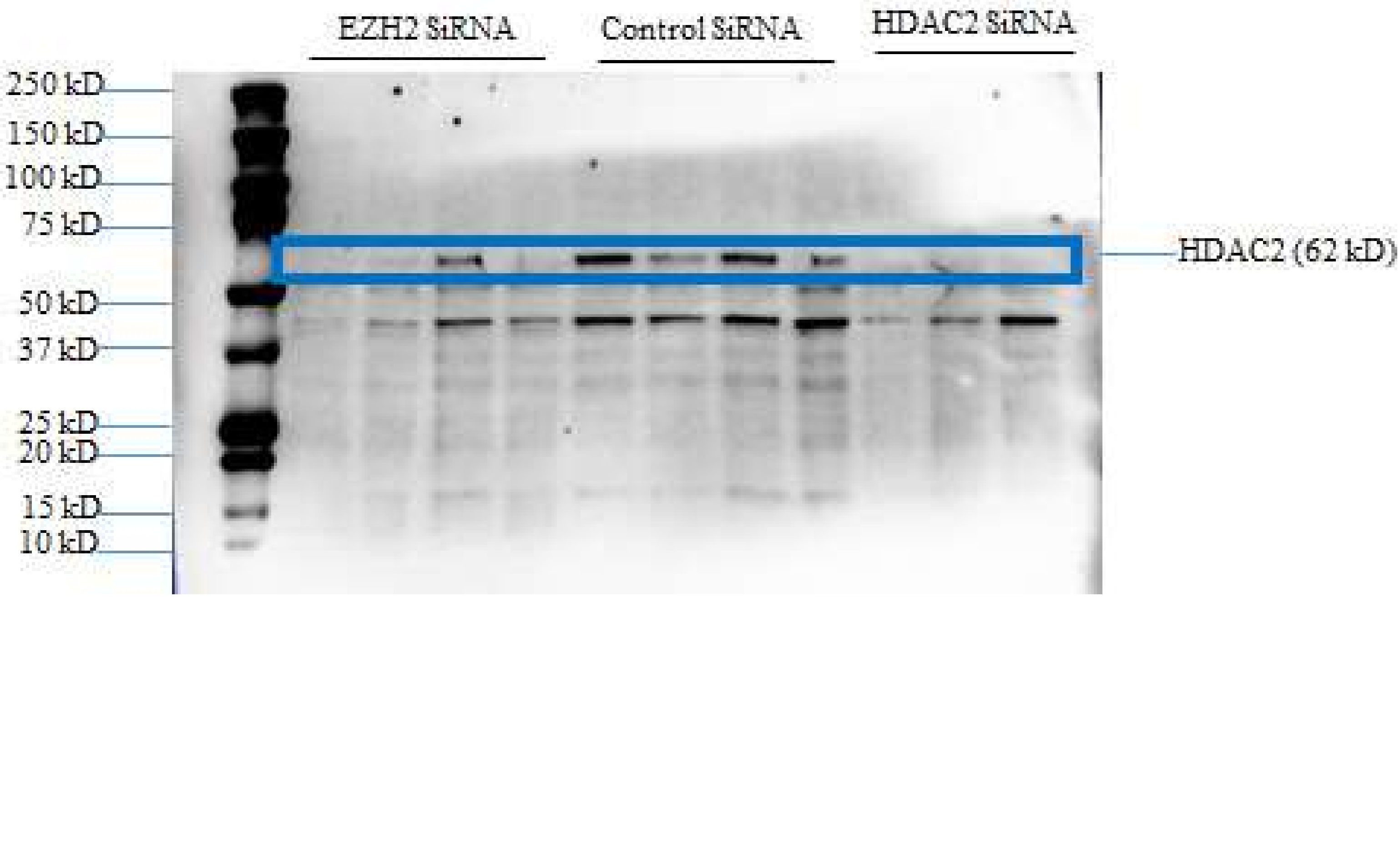

EZH2 SiRNA
Control SiRNA
HDAC2 SiRNA
250 kD
150 kD
100 kD
75 kD
50 kD
37 kD
25 kD
20 kD
15 kD
10 kD
HDAC2 (62 kD)

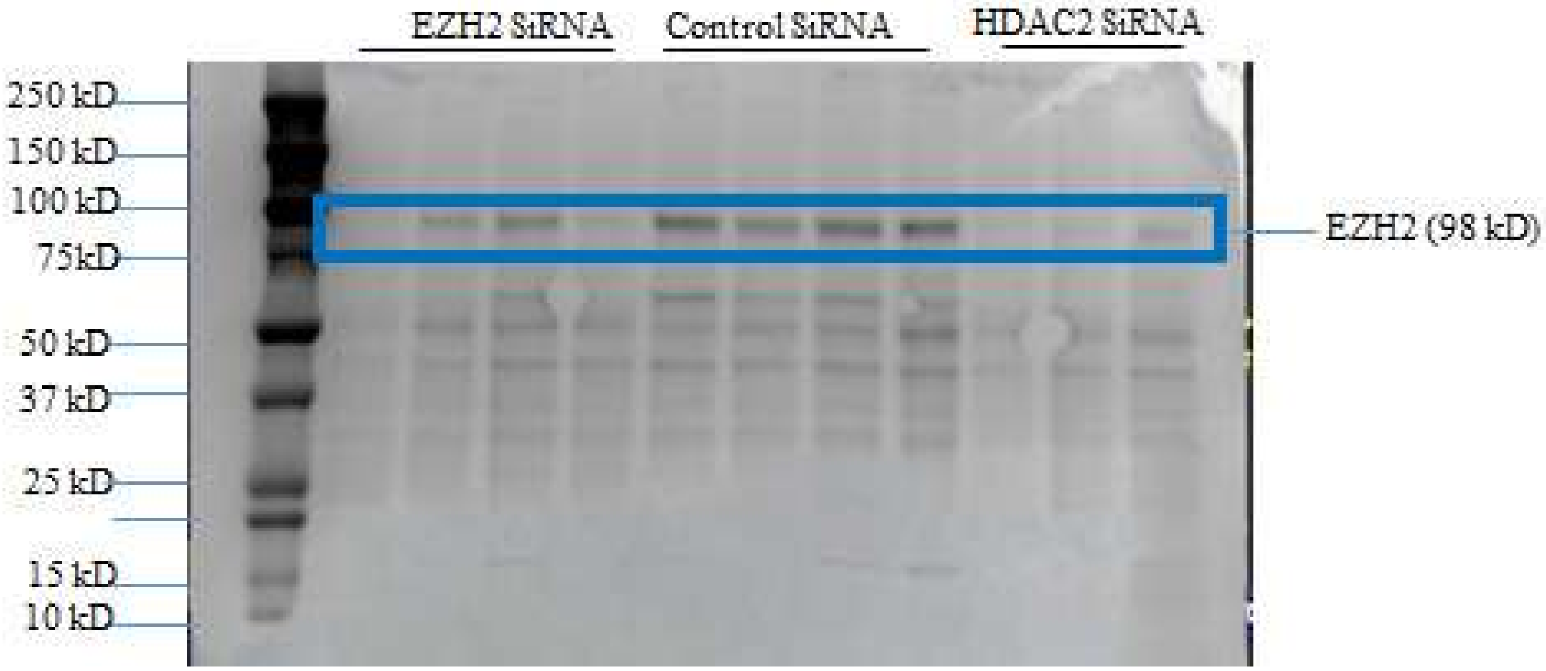
EZH2 SiRNA
Control SiRNA
HDAC2 SiRNA
250 kD
150 kD
100 kD
75kD
50 kD
37 kD
25 kD
15 kD
10 kD
EZH2 (98 kD)
