# Supplementary material for: Key epigenetic and signaling factors in the formation and maintenance of the blood-brain barrier
Source: eLife. 2024 Dec 13;12:RP86978. doi: 10.7554/eLife.86978 (PMC11643625; doi:10.7554/eLife.86978)
Supplement: Supplementary file 1. [file elife-86978-supp1.docx]

| **QPCR Primer** | | | |
| --- | --- | --- | --- |
| **Gene** | **Primer sequence** |  | |
| GAPDH Mouse | FWD AAGGTCATCCCAGAGCTGAA | REV CTGCTTCACCACCTTCTTGA | |
| CLDN1 | FWD ACTCCTTGCTGAATCTGAACAGT | REV GGACACAAAGATTGCGATCAG | |
| CLDN5 | FWD AGCCCCTGTGAAGATTGA | REV TCTGGAGCCTGAGTCTCTG | |
| MFSD2A | FWD CAGGGATCATCGCCTCCATC | REV GAAGGGCATTGACTCAGCCT | |
| ZIC3 | FWD CACGTGCTGGAGGCAATTA | REV GCGAAGGAGATCCCTGAAG | |
| FOXF2 | FWD CCTACTCGTTGGAGCAGAGC | REV TAAGAGCCACTAGCGGAAGG | |
| VEGFA | FWD AACGATGAAGCCCTGGAGTG | REV CTGCTGTGCTGTAGGAAGCT | |
| OCCLUDIN | FWD ACTACCTTGGGTGCTGTGCT | REV AAATTGGGCTGGATGTCAAT | |
| ZO1 | FWD GGGAGGGTCAAATGAAGACA | REV GGCATTCCTGCTGGTTACAT | |
| VEGFA | FWD CACAGCAGATGTGAATGCAG | | REV TTTACACGTCTGCGGATCTT |
| AXIN2 | FWD GAGTAGCGCCGTGTTAGTGACT | | REV CCAGGAAAGTCCGGAAGAGGTATG |
| LEF1 | FWD CAGGAGCCCTACCACGACAA | | REV GCCTCCGTCTGGATGCTTT |
| HDAC1 | FWD CTGTCCGGTATTTGATGGCT | | REV CACGAACTCCACACACTTGG |
| HDAC2 | FWD GGCGGCAAGAAGAAAGTGTGC | | REV GGCATCATGTAGTCCTCCAGC |
| HDAC3 | FWD TCTGAGGACTACATCGACTCC | | REV GTCGCCATCATAGAACTCATTG |
| HDAC8 | FWD GGCTGCGGAACGGTTTTAAG | | REV GCTTCAATCAAAGAATGCACCATAC |
| **Chip-qPCR Primer** | | | |
| CLDN1 TSS | FWD TCTTCAGATGGTCCCCAGGT | | REV CCCGGCGCTGTGATTTAAAG |
| CLDN1 -500 | FWD AGGCACTCTCCGGTTAGCTC | | REV GGGCACCTCTTGTCTGATAGTT |
| CLDN1 +500 | FWD GAACAGTGAGTGCACCCTCA | | REV CAGAGGACAAGGGCTCCAAG |
| MFSD2A TSS | FWD GCGTTCCTGGTTTGCTAAGT | | REV CCTTCTCCTTTGGCCATGA |
| MFSD2A -500 | FWD CTACCCATAAACGGCTGCTG | | REV CAATGTCCCTCGTGTCTCAA |
| MFSD2A +500 | FWD TAAAGGAGCCGCATACCG | | REV ATCGGAGAGAGAGGATGTCG |
| LEF1 TSS | FWD ACCCTCCCTTCTTGCTGTCT | | REV CTCCTCTTCGGGATGACTGA |
| LEF1 -500 | FWD CTGAAGGAAGTGGACTTTTCG | | REV AAACCTCTCCACGGATTCCT |
| LEF1 +500 | FWD CAGGAAACGCTGGCCTATAC | | REV CGGTGGTTTAACGCACTTCT |
| ZIC3 TSS | FWD TGCGAGGAGTGAGTGATTGA | | REV CAACTGCACCAAGAAGCAGA |
| ZIC3 -500 | FWD TGCGGGAATTTGTTCTCGGTA | | REV GTTCCCTTTCGGTCCAGAAGA |
| ZIC3 +500 | FWD CTACGGCCCTTCAGGGATCT | | REV TCCAGGAGCATCGTCATAGGT |
| SOX 17 TSS | FWD GGAGAGGCTAGCAAAGCGAA | | REV GGTCGGAGGTGGAGATGGAA |
| SOX 17 +500 | FWD GCTTAGACTCTCCCACTTCGG | | REV ACTAGAAGCTGTGCCCGTAA |
| SOX 17 -500 | FWD GCAGTCCTACCCAGTTTGCT | | REV GACGGGCTTCCTTGGAGAAA |
| CLDN5 TSS | FWD GGTCTGCTCATGGATTGGTT | | REV ATTGATTCTGGTGCCTCTGC |
| CLDN5 -500 | FWD GTGTGCCAAGGGAAAAACGG | | REV CACCTGCTACATCCCGGATC |
| CLDN5 +500 | FWD GCTGGTGGCACTCTTTGTTA | | REV TAGAACTCGCGGACAACGAT |
| AXIN2 TSS | FWD GAGCGCCTCTGTGATTGG | | REV GCGAACGGCTGCTTATTTT |
| AXIN2 +500 | FWD AGTTTTTGTTGGAAGCGCCC | | REV ACGGAAAACAACGATCCCGA |
| AXIN2 -500 | FWD ACACACACATCATGCCTTCTG | | REV CCCCTTCATCAAATCAAACA |
